# Net present value analysis of total salary compensation in hand surgery: academic versus non-academic careers

**DOI:** 10.1007/s00402-026-06547-2

**Published:** 2026-10-21

**Authors:** John W. Moore, Anthony J. Minerva, Dane N. Daley, Charles A. Daly, Jason Silvestre

**Affiliations:** Medical University of South Carolina, Charleston, USA

**Keywords:** Salary, Compensation, Academia, Surgery, Hand, Orthopaedics

## Abstract

**Purpose:**

Studies comparing salary compensation of hand surgeons in different practice settings have been limited. This study compared projected lifetime salary compensation between academic and non-academic hand surgeons.

**Methods:**

Compensation data were sourced from the 2025 Medical Group Management Association (MGMA) and American Medical Group Association (AMGA) salary benchmarks. Net present values of theoretical career paths in hand surgery were calculated assuming a 32-year career and a 3% discount rate. Sensitivity analyses were performed with variable discount rates and career lengths. Monte Carlo simulations were performed.

**Results:**

Academic practice demonstrated lower lifetime salary compensation than non-academic practice across career trajectories, sensitivity analyses, and Monte Carlo simulations using MGMA data with minimal differences using AMGA data. With MGMA, academic practice ($12.6 million) resulted in 19.7% lower lifetime salary compensation than non-academic practice ($15.7 million). With AMGA, academic practice ($15.0 million) resulted in 2.7% higher lifetime salary compensation than non-academic practice ($14.6 million). Monte Carlo simulations demonstrated lower lifetime salary compensation for academic practice with MGMA ($12.9 million versus $16.0 million, 19.4% difference, *P* < 0.001) but comparable compensation with AMGA data ($15.1 million versus $15.0 million, 0.7% difference, *P* = 0.182).

**Conclusion:**

Hand surgeons in academic practice demonstrated lower lifetime salary compensation than their non-academic counterparts using MGMA data and similar compensation using AMGA data. While compensation alone does not fully capture the complexity of career decision making, this study may be useful to future hand surgeons and service line leaders.

## Introduction

 Orthopaedic surgery is one of the most competitive fields in medicine, which can partially be attributed to favorable salary compensation [1, 2]. Several recent studies have demonstrated that financial compensation in orthopaedic surgery has declined, despite rising inflation and costs of living [3–7]. Currently, hand surgery remains a popular fellowship choice for graduates of orthopaedic surgery residency training programs [8, 9]. However, prior studies have demonstrated that hand surgery fellowship training may be associated with lower lifetime earnings compared to general orthopaedic practice [10, 11]. Therefore, it remains increasingly important for aspiring hand surgeons to understand the financial impactions of their career decisions.

Prior studies have demonstrated that academic surgeons demonstrate lower financial compensation compared to non-academic surgeons [12]. Additionally, a recent analysis found that academic hand surgeon compensation has not kept pace with inflation from 2017 to 2023 [13]. Another study found that academic orthopaedic surgeons had lower total compensation despite increased clinical activity when compared to their non-academic counterparts, although the compensation gap appears to be narrowing [14]. Currently, specific studies comparing salary compensation between academic and non-academic hand surgeons remain limited.

Net present value (NPV) is a financial analysis tool that allows comparison of long-term revenue potential relative to present day purchasing power [15]. This approach may inform hand surgeons on how current career decisions such as pursuing an academic career will affect long-term earning potential. Analysis of NPV values has previously been used to compare lifetime earnings of physicians across several medical specialties [16–19]. However, there has been no study comparing lifetime salary compensation between hand surgeons in academic and non-academic practice. Based on prior research [12], we hypothesized that academic hand surgeons would demonstrate lower lifetime salary compensation than their counterparts in non-academic practice.

## Materials and methods

This study compared lifetime salary compensation between academic and non-academic hand surgeons using NPV modeling and Monte Carlo simulations. Data were sourced from the 2025 Medical Group Management Association (MGMA) and American Medical Group Association (AMGA) salary benchmark surveys. Due to limitations in data share agreements, these datasets included 25th percentile, 50th percentile, and 75th percentile values for both academic and non-academic hand surgeons with orthopaedic surgery training. Salary benchmarks from plastic surgery hand surgeons were not available for comparison. This study adhered to economical study reporting standards from the Consolidated Health Economic Evaluation Reporting Standards (CHEERS) 2022.

Five theoretical career trajectories were simulated in this study:

1. Fixed 25th percentile salary.
2. Fixed median salary.
3. Fixed 75th percentile salary.
4. Standard career: 25th percentile for years 1 to 3, median for years 4 to 10, 75th percentile for years 11 to 20, and median for years 21 to 32.
5. Accelerated career: 25th percentile for years 1 to 2, median for years 3 to 7, 75th percentile for years 8 to 32.

The standard career was based on prior findings that physician earnings typically peak mid-career with a gradual decline thereafter [20]. The accelerated career track modeled a theoretical career in which a hand surgeon reaches higher earnings earlier and does not experience late decline. All NPV calculations were performed using 2024 United Stated Dollars and the standard financial formula:

$$ NPV = \:\sum\limits_{{t = 1}}^{N} {\frac{{S_{t} }}{{\left( {1 + r} \right)^{t} }}} $$

Where:

- N = career duration (years).
- t = salary year.
- S_t_ = salary in year t.
- r = discount rate.

The primary study outcome was lifetime salary compensation for a 32-year career, assuming retirement age for orthopaedic surgeons of 65 years old, which is consistent with prior surveys completed by the American Academy of Orthopaedic Surgeons [21]. The base model used a 3% annual discount rate. Sensitivity analyses were completed using varying career lengths (28, 30, 32, 34, and 36 years) and discount rates (1–5%).

A Monte Carlo simulation was then performed to model uncertainty in projected lifetime salary compensation. A total of 10,000 iterations were conducted for each practice setting. Each iteration randomly selected: (1) a fixed salary of 25th percentile, median, or 75th percentile, (2) a random discount rate between 1 and 5%, and (3) a random integer career length between 28 and 36 years. Resulting NPVs for each practice setting were summarized using medians and interquartile ranges. Figure generation and statistical analysis were performed using Microsoft Excel version16.108.2 (Microsoft Corporation, Redmond, WA, USA) and R version 4.3.2 (R Foundation for Statistical Computing, Vienna, Austria), respectively. Monetary values were reported in millions of 2024 United States Dollars. Results from the Monte Carlo simulations were reported as median NPVs with interquartile ranges (IQRs). Mann Whitney U tests were used to compare median NPVs resulting from Monte Carlo simulations with an alpha set at 0.05 for statistical significance.

## Results

### Results of net present value calculations

**Fig. 1:**
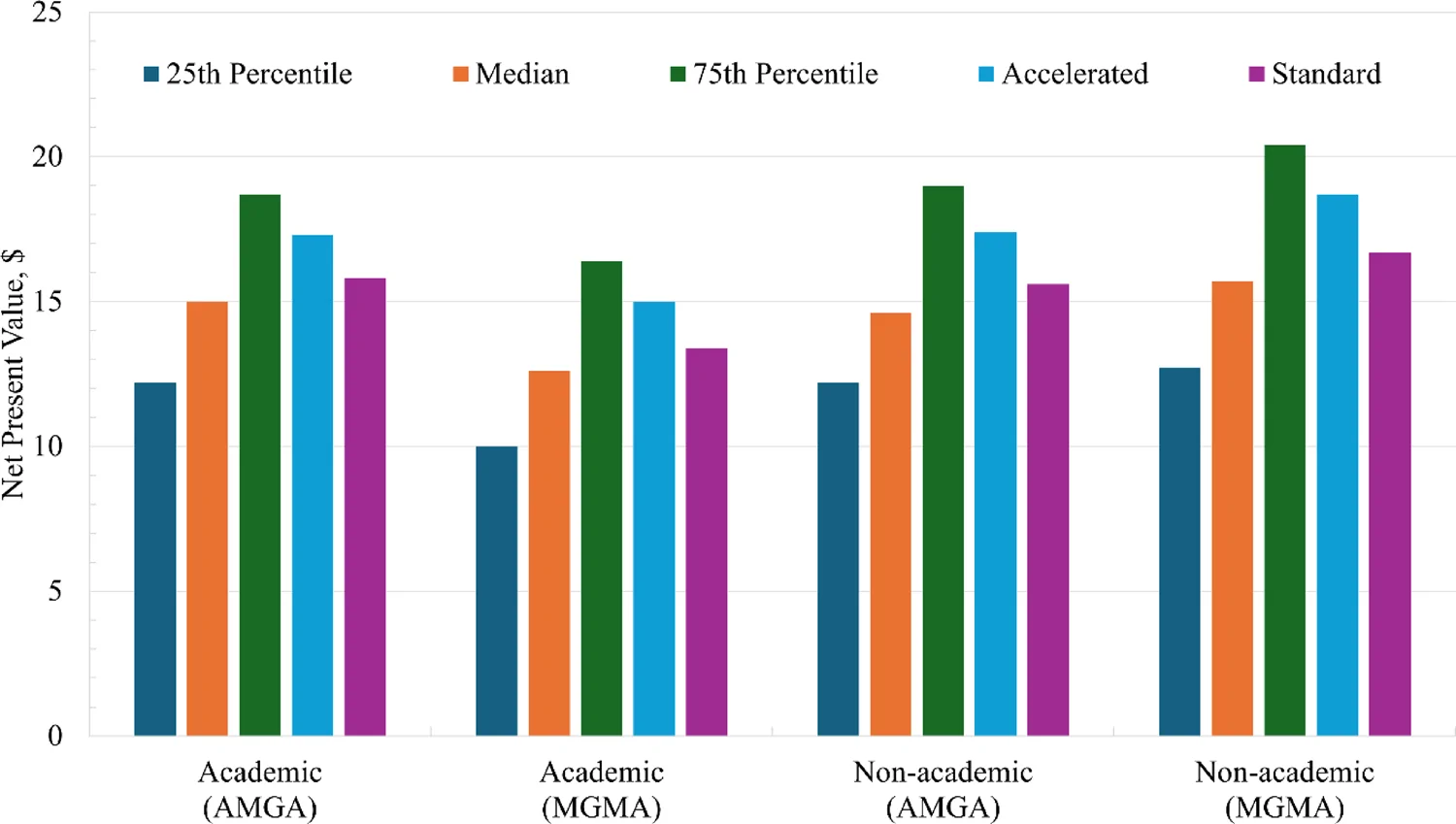
Net present value of lifetime salary compensation across five simulated career trajectories. Net present values presented in millions of dollars; 25th: fixed 25th percentile compensation over the course of a simulated career; Median: fixed 50th percentile compensation over the course of a simulated career; 75th: fixed 75th percentile compensation over the course of a simulated career; Standard: variable compensation at the 25th percentile for years 1 to 3, median for years 4 to 10, 75th percentile for years 11 to 20, and median for years 21 to 32.; Accelerated: variable compensation at the 25th percentile for years 1 to 2, median for years 3 to 7, and 75th percentile for years 8 to 32; *MGMA* Medical Group Management Association, *AMGA* American Medical Group Association, *USD* United States Dollars

Academic practice models demonstrated lower lifetime salary compensation than non-academic practice models across all five simulated career trajectories under MGMA benchmarks, while differences were minimal under AMGA benchmarks (Fig. 1). With MGMA benchmarks, academic practice demonstrated lower lifetime salary compensation than non-academic practice for fixed salaries at the 25th percentile ($10.0 million vs. $12.7 million, 21.3% difference), median ($12.6 million vs. $15.7 million, 19.7% difference), and 75th percentile ($16.4 million vs. $20.4 million, 19.6% difference), as well as standard ($13.4 million vs. $16.7 million, 19.8% difference) and accelerated ($15.0 million vs. $18.7 million, 19.8% difference) career trajectories. With AMGA benchmarks, lifetime salary compensation was similar between academic and non-academic practice across all career trajectories, with differences of less than 3% across all comparisons (range, 0.0–2.7% difference).

### Impact of discount rates on lifetime salary compensation

**Fig. 2:**
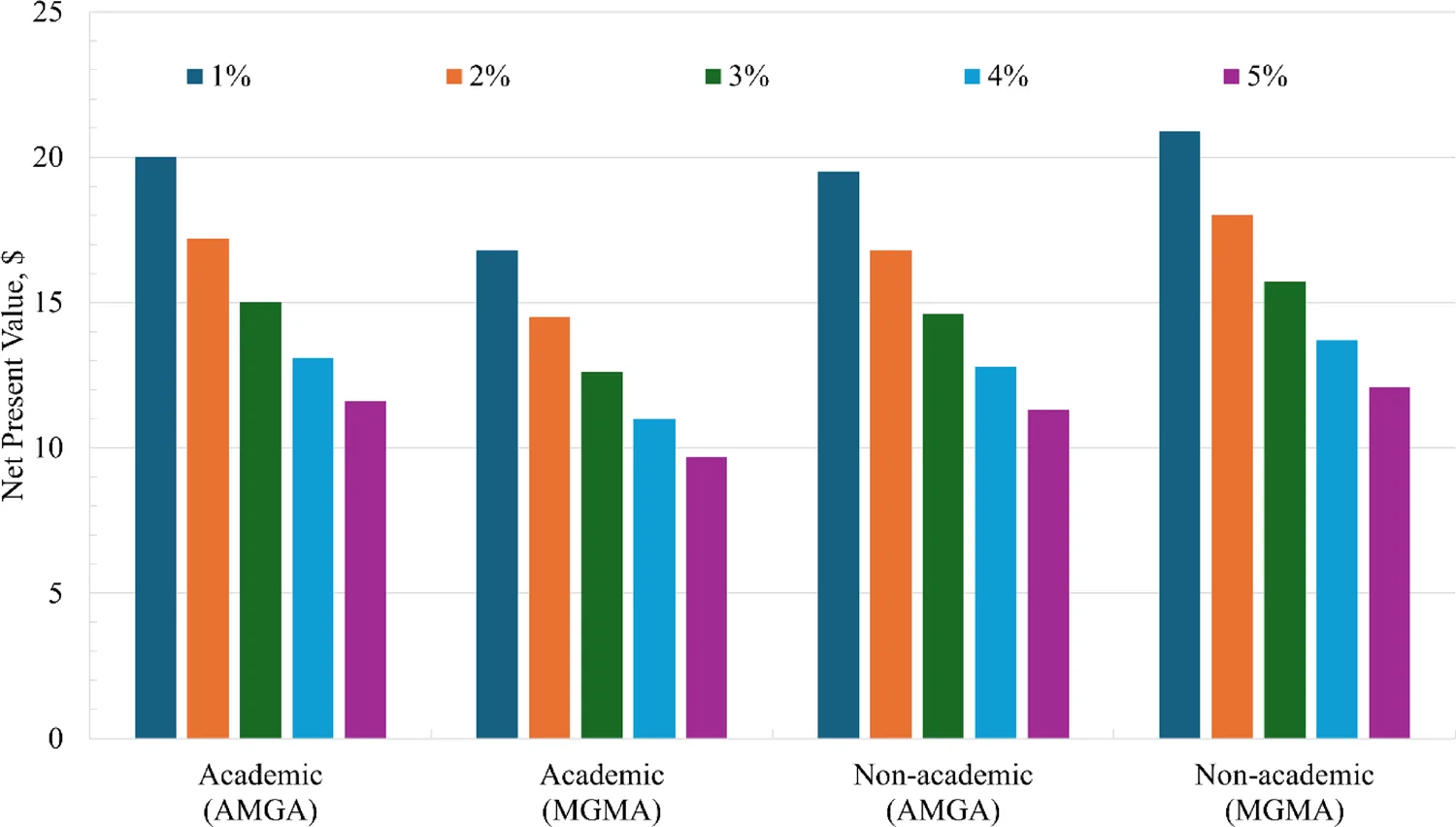
Net present value of lifetime salary compensation across five different discount rates. Net present values presented in millions of dollars; discount rates included 1%, 2%, 3%, 4%, and 5%; *MGMA* Medical Group Management Association, *AMGA* American Medical Group Association, *USD* United States Dollars

Lifetime salary compensation assuming a fixed median salary and a 32-year career length was compared between academic and non-academic practice settings across varying discount rates (Fig. 2). With MGMA, academic practice demonstrated lower lifetime salary compensation than non-academic practice across discount rates, ranging from $9.7 million to $16.8 million compared with $12.1 million to $20.9 million (range, 19.4–19.8% difference). With AMGA, academic practice and non-academic practice demonstrated similar lifetime salary compensation, ranging from $11.6 million to $20.0 million compared with $11.3 million to $19.5 million (range, 2.3–2.7% difference).

### Influence of career length on lifetime salary compensation

**Fig. 3:**
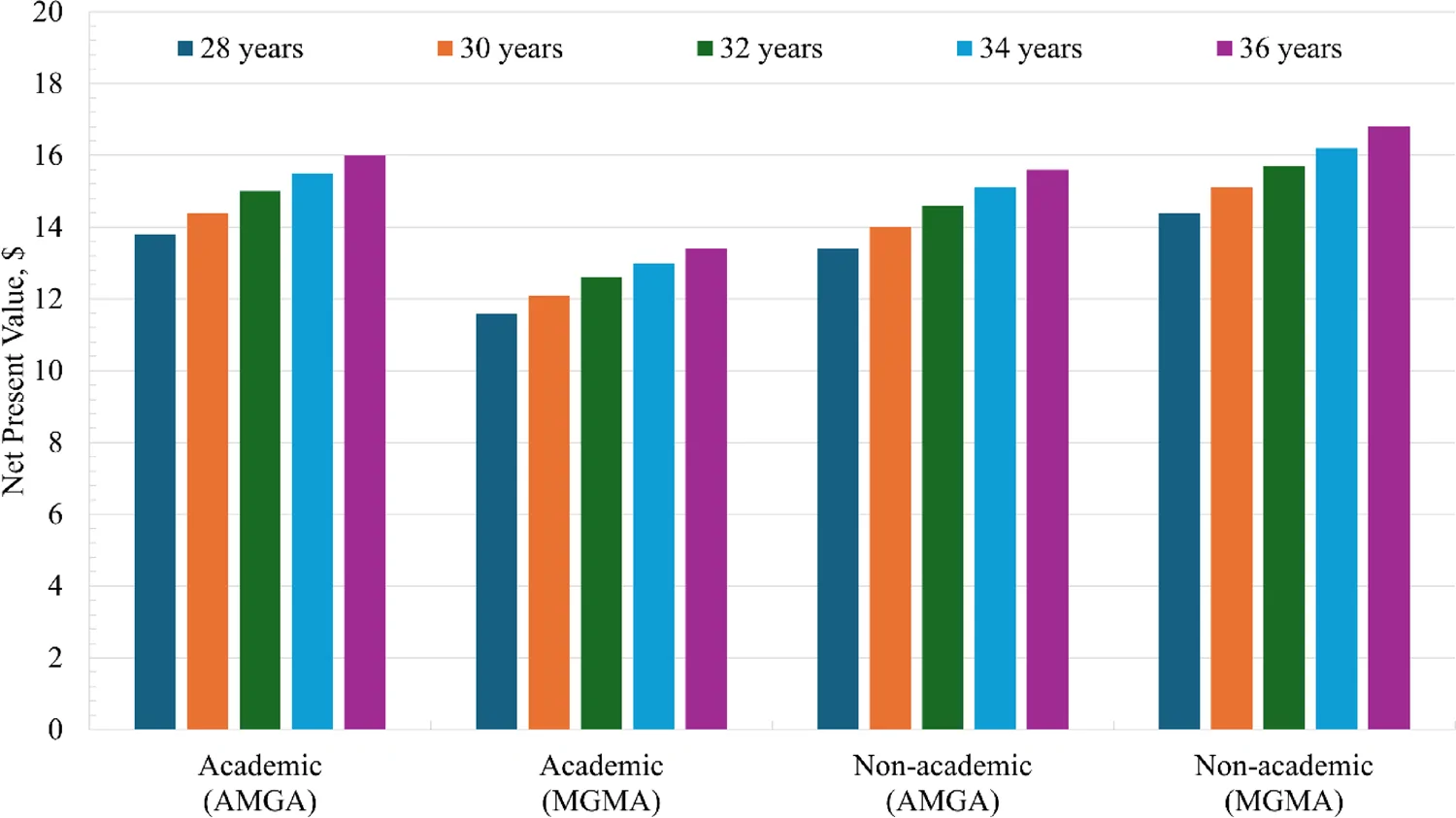
Net present value of lifetime salary compensation across five simulated career lengths. Net present values presented in millions of dollars; career lengths included 28, 30, 32, 34, and 36 years; *MGMA* Medical Group Management Association, *AMGA* American Medical Group Association, *USD* United States Dollars

Lifetime salary compensation assuming a fixed median salary and a 3% discount rate was compared between academic and non-academic practice across five simulated career lengths (Fig. 3). With MGMA, academic practice demonstrated lower lifetime salary compensation than non-academic practice across all career lengths, ranging from $11.6 million to $13.4 million for academic practice compared with $14.4 million to $16.8 million for non-academic practice (range, 19.4–20.2% difference). With AMGA, academic and non-academic practice lifetime salary compensation were similar across all career lengths, ranging from $13.8 million to $16.0 million for academic practice compared with $13.4 million to $15.6 million for non-academic practice (range, 2.6–3.0% difference).

### Results from Monte Carlo simulations

**Fig. 4:**
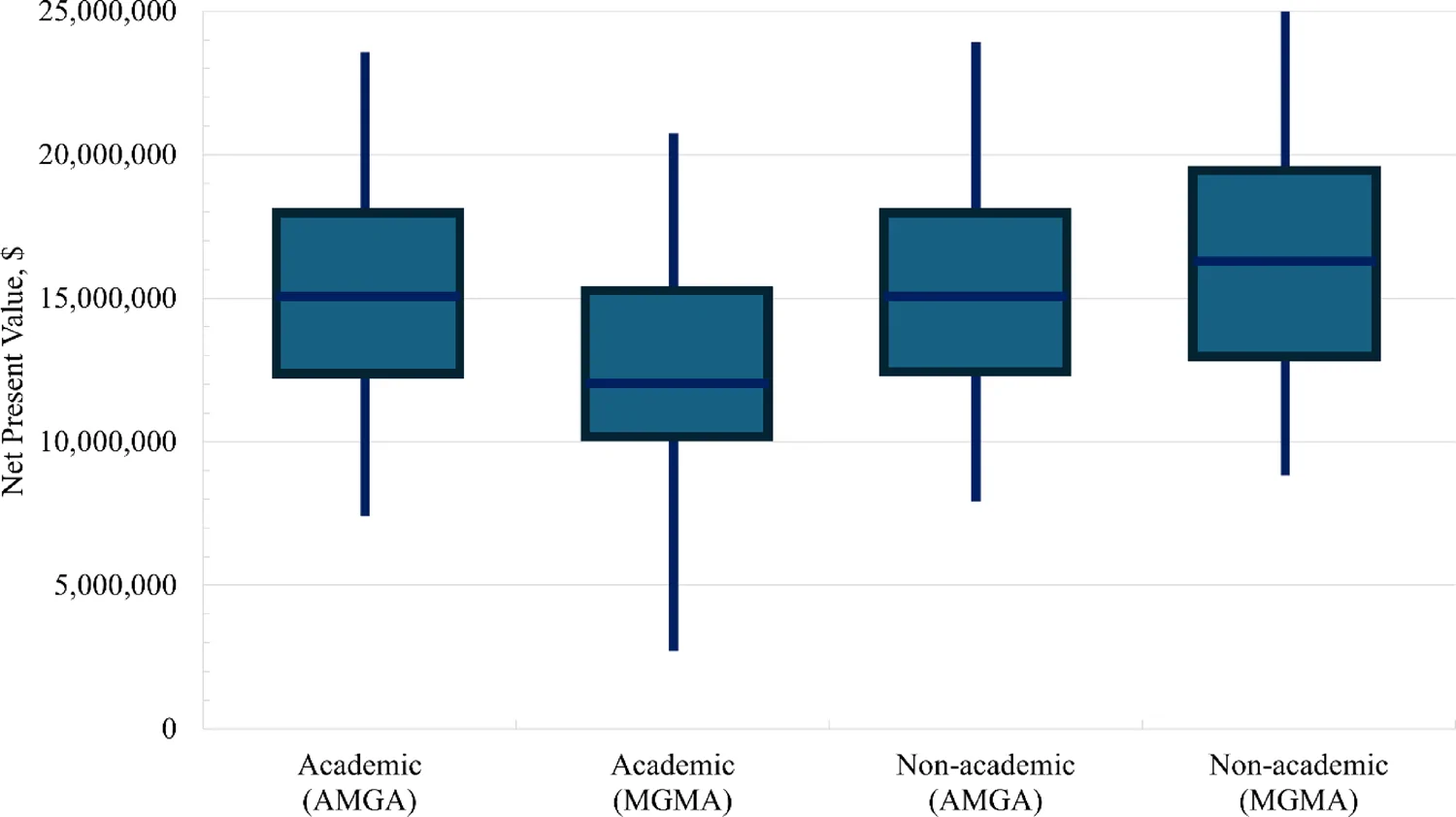
Box and whisker plots demonstrating results from a Monte Carlo simulation for each practice model. Box and whisker plot with the horizontal line corresponding to the median with ends of boxes corresponding to the 25th and 75th percentiles and end of lines corresponding to the minimum and maximum; *MGMA* Medical Group Management Association, *AMGA* American Medical Group Association, *USD* United States Dollars

Monte Carlo simulations were performed for each of the four practice models (Fig. 4). With MGMA, academic practice had lower lifetime salary compensation than non-academic practice ($12.9 million, IQR $10.5–$15.4 million versus $16.0 million, IQR $13.3–$19.2 million, *P* < 0.001). With AMGA, academic practice had similar lifetime salary compensation than non-academic practice ($15.1 million, IQR $12.6–$17.9 million versus $15.0 million, IQR $12.4–$17.7 million, *P* = 0.182).

## Discussion

Non-academic practice MGMA benchmarks consistently result in the highest lifetime salary compensation across all comparative career scenarios, sensitivity analyses, and Monte Carlo simulations. In contrast, academic practice MGMA benchmarks resulted in the lowest lifetime salary compensation across all scenarios. However, AMGA benchmarks resulted in similar lifetime salary compensation between academic and non-academic practices. Differences in survey methodology between data sources including sampling and practice representation may account for these differences. Large healthcare organizations with a greater representation of hospital employed physicians tend to concentrate in AMGA data relative to MGMA [22], which may help explain similar salary compensation observed between academic and non-academic practice hand surgeons in AMGA data. In contrast, the MGMA survey may sample a larger representation of hand surgeons in non-academic practice that have access to ancillary practice revenue [23]. Regardless of these differences, data from this study may be useful to trainees and junior faculty during career planning, recruitment, and contract negotiation. Furthermore, these results may have implications on academic workforce planning efforts in hand surgery including faculty development and retention.

High rates of attrition and issues with faculty retention may challenge academic, teaching, and research missions of academic orthopaedic departments [24]. Large compensation disparities may negatively impact recruitment efforts of hand surgery faculty especially as financial pressures continue to rise [25]. Academic institutions seeking to attract the best hand surgeons may need to consider additional strategies that better align financial compensation with the diverse contributions of academic surgeons. Such approaches could include enhanced financial incentive structures, protected research time, loan repayment programs, leadership development opportunities, or recognition of educational achievements within existing compensation structures [26]. Importantly, results from this study highlight only one dimension of career value and should be interpreted within the broader context of professional fulfillment, institutional support, and lifestyle considerations in hand surgery. Academic surgeons are likely to see increases in lifetime earnings with academic promotions, including from assistant to associate professor [27]. However, academic promotion is often contingent on research productivity, service line leadership, and national recognition among other professional requirements. Hybrid academic compensation models, which combine a base salary with incentives for case volume and academic contributions may offer greater career flexibility but are often accompanied by more administrative tasks [26]. Furthermore, hand surgeons in academic practice often have greater access to industry funding sources including consulting and research arrangements [28].

A recent survey of orthopaedic surgeons showed academic surgeons were more satisfied with their research opportunities, reputation, and continued education, but dissatisfied with their ancillary income opportunities [29]. In contrast, orthopaedic surgeons in private practice were more satisfied with their autonomy, salary, and ancillary income opportunities, but dissatisfied with their research and continued education opportunities. A survey by the American Society for Surgery of the Hand found high rates of job satisfaction across practice settings despite a high prevalence of burnout [30]. Lower rates of burnout for hand surgeons were found in those not taking call, regardless of compensation for call [30]. Hand surgeons may have lower rates of burnout and depression than their peers in other orthopaedic subspecialties, but hand surgery does not rank among the lowest burnout orthopaedic subspecialties [31]. Importantly, dissatisfaction with salary compensation is often cited as a major source of job attrition in orthopaedic surgery [32]. In our study, there were minimal difference between lifetime salary compensation between academic and non-academic practice using AMGA data. This may be due to the source of AMGA surveys, which tend to focus on large healthcare organizations, thereby skewing responses in favor of practices owned by health systems [33]. With shifting methods of remuneration and the general decline of orthopaedic surgeon reimbursement, the traditional private practice model is becoming less profitable [34, 35]. A large increase in vertical integration of private practices and decreases in fee-for-service payments are driving factors in declining salary compensation [36]. Vertical integration of traditional private practices under large health systems combined with tighter budgets driven by decreasing reimbursement could drive future reimbursement for academic and private practice hand surgeons closer together. Previous studies have also shown disparities in compensation between male and female orthopaedic surgeons [37]. Additional subgroup analysis into compensation differences could improve understanding of the factors that affect lifetime salary compensation. Future research should evaluate additional sources of income including industry payments, consulting fees, practice equity, and benefits across practice settings and subspecialties.

Several limitations should be considered when interpreting results from this study. While salary benchmarks were sourced from the most common sources, both AMGA and MGMA surveys are performed in a voluntary and nonrandom manner. As such, these results may not fully capture regional variabilities, practice specific differences, or reimbursement changes over time. Projections of lifetime salary compensation rely on base assumptions regarding salary growth, retirement age, and inflation. Variations in these parameters could alter absolute salary compensation estimates although relative differences between practice settings may remain similar. Any number of policy or reimbursement shifts can occur over a thirty-year career in hand surgery, which may affect the financial dynamic between non-academic and academic practice. In this study, financial compensation represented only direct salary compensation including ancillary revenue streams, which may not fully capture equity ownership, consulting activities, industry relationships, or other sources of income. Additionally, this study did not account for debt repayment, cost-of-living adjustments, or non-salary benefits. Lastly, non-financial metrics such as career satisfaction, burnout, and work-life integration were not assessed and warrant future investigation.

In conclusion, non-academic practice demonstrated higher lifetime salary compensation than academic practice when using MGMA benchmarks for hand surgeons. However, there was similar lifetime salary compensation between non-academic practice and academic practice when using AMGA benchmarks for hand surgeons. These findings provide valuable insight regarding the financial implications of career choice, which may help assist future hand surgeons during career planning and recruitment. However, compensation represents only one aspect of a hand surgeon’s professional trajectory. Decisions regarding career choice should balance financial considerations with professional interests, educational commitments, research aspirations, and personal goals.

## Data Availability

No datasets were generated or analysed during the current study.
